# iTRAQ-based protein profiling and functional identification of four genes involved in rice basal resistance against *Magnaporthe oryzae* in two contrasting rice genotypes

**DOI:** 10.1007/s44154-023-00118-w

**Published:** 2023-09-12

**Authors:** Chenchen Li, Ziqiang Chen, Yun Deng, Shuyu Jiang, Yan Su, Shaohua Yang, Yan Lin, Dagang Tian

**Affiliations:** 1 Biotechnology Research Institute, Fujian Academy of Agricultural Sciences, Fuzhou, 350003 Fujian China; 2 College of Agriculture, College of Life Science, Fujian Agricultural and Forestry University, Fuzhou, 350002 China; 3 Nanping Institute of Agricultural Sciences, Fujian, China

**Keywords:** Rice, iTRAQ, CRISPR/Cas9, Rice blast, Basal resistance gene

## Abstract

Rice blast, caused by *Magnaporthe oryzae*, is one of the most destructive rice diseases. Developing blast-resistant rice cultivars represents the most economical and environmentally friend strategy for managing the disease. In our previous study, an isobaric tags for relative and absolute quantitation (iTRAQ)-based comparative protein quantification was carried out to investigate the resistance gene *Piz-t* gene-mediated resistance response to infection in two contrasting rice genotypes of the *Piz-t* transgenic Nipponbare line (NPB-Piz-t) and its wild-type Nipponbare (NPB). Here, from the comparisons of differentially expressed proteins (DEPs) of NPB-Piz-t to the avirulent isolate KJ201 (KJ201-Piz-t)and the virulent isolate RB22 (RB22-Piz-t) with mock-treated NPB-Piz-t (Mock-Piz-t), NPB to the virulent isolate KJ201(KJ201-NPB) and RB22 (RB22-NPB) with mock-treated NPB (Mock-NPB), 1, 1, and 6 common DEPs were, respectively, identified at 24, 48 and 72 h post-inoculation (hpi) in the susceptible comparisons of RB22-Pizt/Mock-Piz-t, KJ201-NPB/Mock-NPB, and RB22-NPB/Mock-NPB, involving in gi|54,290,836 and gi|59,800,021 were identified in the resistance comparison KJ201-Piz-t/Mock-Piz-t at 48 and 72 hpi respectively. Moreover, four genes of Os01g0138900 (gi|54,290,836), Os04g0659300 (gi|59,800,021), Os09g0315700 (gi|125,563,186) or Os04g0394200 (gi|21,740,743) were knocked out or overexpressed in NPB using gene over-expression and CRISPR/Cas9 technology, and results verified that the Os01g0138900 obviously affected the rice blast resistance. Further, expression and targeted metabolomics analysis illuminated the resistance response of cysteine-containing substances as gi|59,800,021 under blast infection. These results provide new targets for basal resistance gene identification and open avenues for developing novel rice blast resistant materials.

**Supplementary Information:**

The online version contains supplementary material available at 10.1007/s44154-023-00118-w.

## Introduction

Rice (*Oryza sativa* L.) is one of the most important staple foods feeding nearly 60% population worldwide. However, its production and quality are severely threatened by a variety of pathogens, such as *Magnaporthe oryzae* (*M. oryzae*), is the most notorious destructive pathogens, causing rice blast (Deng et al., 2023). The most economic and effective approach to control the diseases is to create the rice cultivars with broad-spectrum resistance (*R*) gene (He et al. 2022).

Plants defend against pathogens via innate immune responses, which are initiated by cell surface pattern recognition receptors (PRRs) and intracellular nucleotide-binding domain leucine-rich repeat containing receptors (NLRs) leading to pattern-triggered immunity (PTI) and effector-triggered immunity (ETI), respectively (Dangl et al. 2013). PTI and ETI involve different activation mechanisms and many similar downstream responses. In addition, PRR-mediated and NLR-mediated signalling cascades are interdependent and function synergistically to provide robust resistance against pathogens (Yuan et al. 2021). In reality, PTI and ETI share central hubs of signaling including ROS burst, MAPK cascades, and activation of PR genes (Tsuda and Katagiri 2010; Yu et al. 2017). Some studies indicates that cysteine-rich receptor-like kinases (CRKs) and cysteine-containing metabolities such as glutathione are involved in regulating both PTI and ETI responses (Lu et al. 2023).

Recently, more than 100 major rice blast *R* genes have been identified, among which 37 have been cloned and characterized (Yin et al. 2021). Except for *Pid2*, *pi21* and *Ptr*, most known *R* genes encode typical NLR domain, which directly or indirectly interact with fungal effectors to trigger ETI (Dangl et al. 2013; Yin et al. 2021). Although r *R* genes confer a broader-spectrum resistance to rice blast, the level of resistance is not adequate to prevent significant crop losses, easy to be broken down due to the presence of the high variability of the fungal population(Niks et al. 2015; Zhu et al. 2016). Thus, more durable control of rice blast is urgently needed, and a better understanding of non-host or basal resistance may offer opportunities in breeding for sustainable disease control. This form of disease resistance exhibited by all members of a plant species to all genetic variants of a non-adapted pathogen species is known as basal resistance. Due to its broad-spectrum effectiveness and durability, basal resistance is of considerable interest for crop resistance improvement (Zhao et al. 2016).

The rice blast *R* gene *Piz-t*, which encodes a nucleotide-binding site-leucine-rich repeat protein, is a member of the *Pi2/9* multi-allelic gene family (Zhou et al. 2006). *Piz-t* corresponds to the *M. oryzae* avirulence (Avr) gene AvrPiz-t in a gene-for-gene fashion (Li et al. 2009). Studies have revealed that the interaction of Piz-t and AvrPiz-t involves two rice ubiquitin E3 ligases of APIP6 (AvrPiz-t Interacting Protein 6) and APIP10, bZIP-type transcription factor APIP5 and its Nup98 family protein APIP12, and K + channel protein OsAKT1 APIP7 to suppress host basal defense (Park et al. 2012, 2016; Wang et al. 2016; Tang et al. 2017; Zheng et al. 2023). Further, APIP5 directly interacts with OsWAK5 and CYP72A1, which play roles in ROS production and defense compound accumulation, respectively (Younas et al., 2023). These studies also suggest that *Piz-t* and many basal *R* genes are indispensable for resistance response (Younas et al., 2023).

Our previous study identified several DEPs by using isobaric tags for relative and absolute quantitation (iTRAQ)-based protein profiling (Tian et al. 2018). However, those comparisons have been only carried out to preliminary investigate the Piz-t-mediated resistance loci. As CRISPR/Cas9 has been exploited as a robust, accurate, efficient and programmable method for genome targeting and editing, the mutation or down-regulation of susceptibility (*S*) genes that could confer a non-race-specific and potentially durable resistance have been widely applied for resistance improvement (Van Schie and Takken 2014). For example, knocking out either of *S* genes *Bsr-d1*, *Pi21*, and *ERF922* (Yasuda et al. 2015; Wang et al. 2016; Li et al. 2017) can enhance resistance to rice blast or rice bacterial leaf blight disease. Nevertheless, only a few S genes have been applied in rice breeding (Van Schie and Takken 2014).

In this study, we re-analysed the iTRAQ data. By widely comparison, eight common DEPs were identified in the susceptible comparisons of RB22-Pizt/Mock-Piz-t, KJ201-NPB/Mock-NPB, and RB22-NPB/Mock-NPB, including two in resistance comparison of KJ201-Pizt/Mock-Piz-t. By using gene over-expression and CRISPR/Cas9 technology, Os01g0138900 was identified to be associated with rice blast resistance. Furthermore, gene expression and targeted metabolomics analyses indicated that Os04g0659300 and its downstream proteins with cysteine residues were activated under rice blast infection. Therefore, our results provide a new strategy for candidate basal *R* gene identification and pave the way for the development of novel rice blast-resistant materials.

## Results

### Common differentially expression proteins in NPB-Piz-t and NPB in response to *M. oryzae* infection

**Fig. 1:**
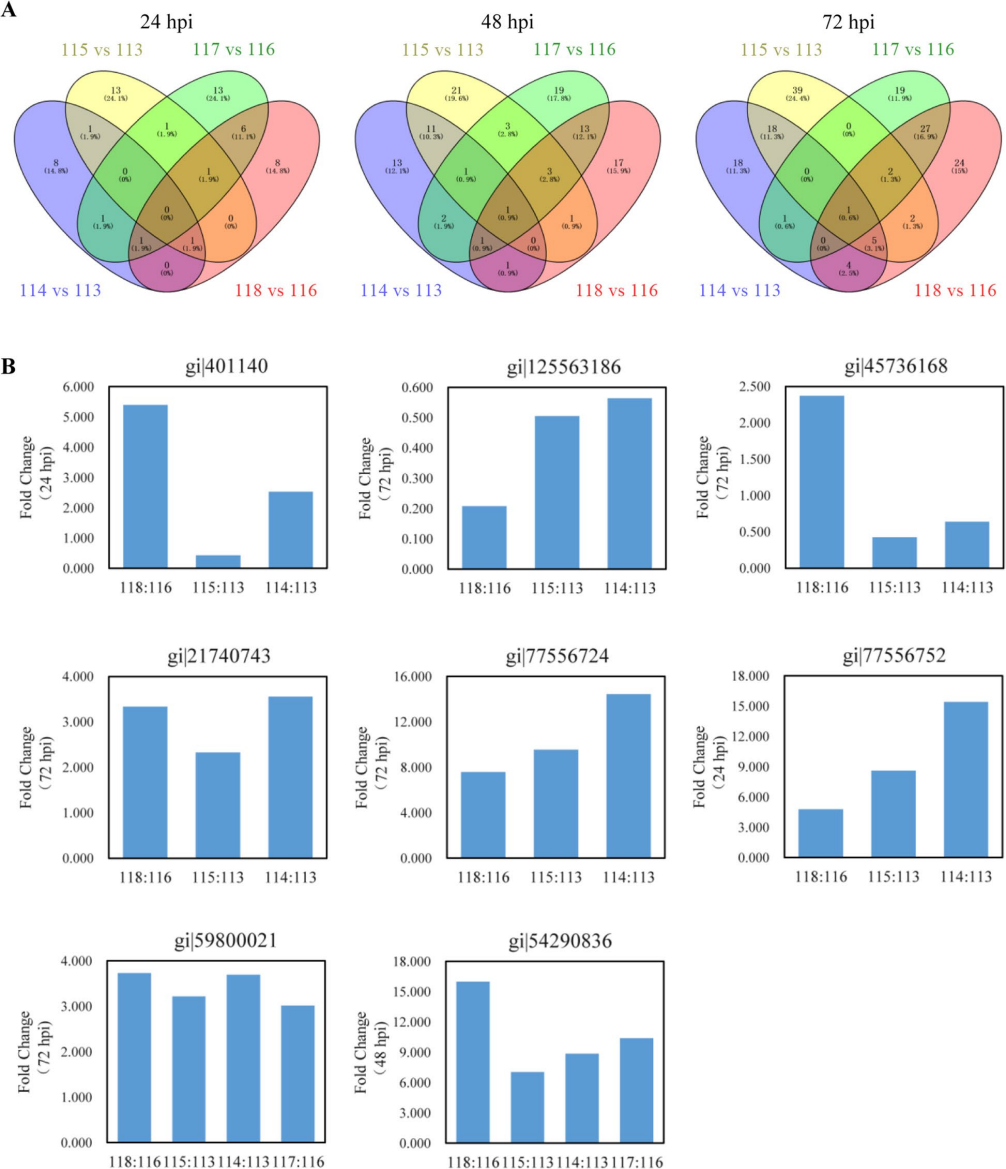
Venn diagrams and expression patterns of differentially expressed proteins in the iTRAQ identified rice blast basal resistance-associated loci. **a** Venn diagrams of differentially expressed proteins in the four comparisons. **b** Five types of expression patterns in the 8 proteins, 1, 1, 1, 3, 2 proteins in type 1 to type 5, respectively.114:113, KJ201-NPB/Mock-NPB; 115:113, RB22-NPB/Mock-NPB; 117:116, KJ201-Piz-t/Mock-Piz-t; 118:116, RB22-Piz-t/Mock-Piz-t

We re-analyzed the iTRAQ data of NPB-Piz-t and NPB in response to the avirulent or virulent isolate KJ201 or RB22 (Tian et al. 2018). Only 1, 1, and 6 proteins were identified to be the common DEPs in the comparisons of RB22-Pizt/Mock-Piz-t, KJ201-NPB/Mock-NPB, and RB22-NPB/Mock-NPB at 24, 48, and 72 hpi, among which two proteins gi|54,290,836 and gi|59,800,021 were identified in KJ201-Pizt/Mock-Piz-t at 48 and 72 hpi respectively (Fig. 1, Table 1). The expression patterns of those eight proteins were classified five types. Expression patters of gi|401,140 in type 1 were up-regulated in RB22-Pizt/Mock-Piz-t and KJ201-NPB/Mock-NPB, but down-regulated in RB22-NPB/Mock-NPB (Fig. 1). Type 2 indicated that the expression level of gi|125,563,186 in susceptible response much lower than in their corresponding CK, Type 3 exhibited that gi|45,736,168 has an differential expression patterns in RB22-Pizt/Mock-Piz-t, KJ201-NPB/Mock-NPB, and RB22-NPB/Mock-NPB, with up-, down- and down-regulated, respectively. Type 4 included gi|21,740,743, gi|77,556,724, and gi|77,556,752 that exhibited significance higher in the susceptible response than in their corresponding CK. Type 5 contained gi|59,800,021 and gi|54,290,836, indicating that the expression level were higher in resistance and susceptible than their corresponding CK. We speculate that rice and *M.oryzae* interaction might activate or suppress these basal *R* genes to resist or speed up rice blast disease.

**Table 1:** The information of eight differentially expressed proteions in NPB-Piz-t and NPB upon *M.oryzae* infection

GI	Gene symbol	description
gi|401,140	Os03g0401300	Sucrose synthase 2, plasma membrane-localized
gi|125,563,186	Os09g0315700	putative phosphoenolpyruvate carboxylase
gi|45,736,168	Os08g0560900	putative photosystem I reaction center subunit II, chloroplast precursor
gi|21,740,743	Os04g0394200	dihydrolipoyllysine-residue succinyltransferase
gi|77,556,724	Os12g0629700	thaumatin-like proteins
gi|77,556,752	Os12g0555200	pathogenesis-related protein Bet v I family protein
gi| 59,800,021	Os04g0659300	cysteine-rich receptor-like protein kinases
gi|54,290,836	Os01g0138900	L-Ala-D/L-amino acid epimerase

### Validation of the types 2, 4 and 5 proteins functions via over-expression and CRISPR/Cas9 technology

To confirm whether those DEPs were correlated with rice blast resistance, their functions were firstly analysed. Type 1 with a plasma membrane-localized protein (gi|401,140), and type 2 contained gi|125,563,186 was annotated as putative phosphoenolpyruvate carboxylase, and type 3 was putative photosystem I reaction center subunit II, chloroplast precursor (gi|45,736,168).Type 4 contained dihydrolipoyllysine-residue succinyltransferase (gi|21,740,743) involved in TCA cycle, two pathogenesis-related proteins of pathogenesis-related protein Bet v I family protein (gi|77,556,752) and thaumatin-like proteins (gi|77,556,724). Additionally, type 5 included cysteine-rich receptor-like protein kinases(gi|59,800,021) and L-Ala-D/L-amino acid epimerase (gi|54,290,836).

**Fig. 2:**
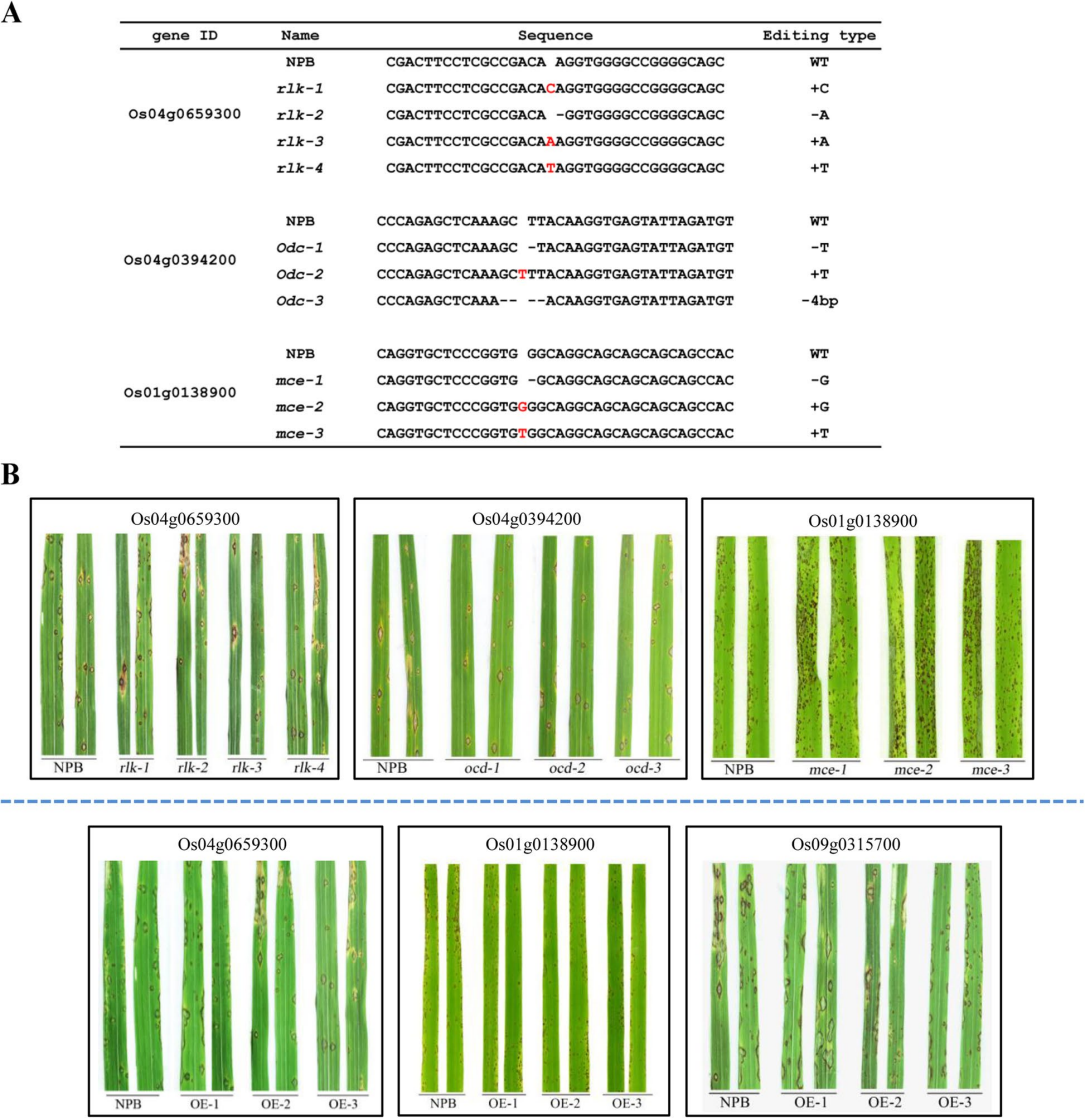
CRISPR-Cas9 edited and rice blast disease evaluation of accessions carrying knock-outs and over-expression. **a** Editing type of the mutants of Os04g0659300, Os04g0394200, and Os01g0138900. **b** Spray inoculation of accessions with different knock-outs of Os04g0659300, Os01g0138900, and Os04g0394200 with rice blast strain of KJ201. **c** Spray inoculation of accessions with different over-expression of Os04g0659300, Os01g0138900 and Os09g0315700, with rice blast strain of KJ201

As inconsistent expression patterns of type 1 and 3 existed among those susceptible groups (Fig. 1), so we selected three genes from type 4 and 5 using CRISPR/Cas9 technology to investigate the contribution of those proteins to rice blast resistance. Using NPB as the background material and three genes of Os04g0394200 (gi|21,740,743), Os04g0659300 (gi|59,800,021), and Os01g0138900 (gi|54,290,836) for editing. For Os04g0659300, we obtained four editing types, they were insertion of “T”, “A” or “C” and a “A” deletion (Fig. 2). For Os04g0394200, three editing types were obtained, including a “4 bp”and a “T”deletion, a “TT” insertion (Fig. 2a). Further, a “G”, “T” insertion and a “G” deletion were obtained for Os01g0138900 (Fig. 2a). Spray inoculation results showed that mutants of Os01g0138900 were more susceptible to rice blast, while mutants of Os04g0394200 and Os04g0659300 were similar to that of the wild type (WT) (Fig. 2b).

We have demonstrated that Os01g0138900 influence rice blast resistance. In addition, expression patterns of gi|125,563,186 and gi|59,800,021 were consistent in susceptible or resistance response, suggesting that they should have relations with rice blast resistance. To confirm this hypothesis, we over-expressed these genes in the NPB background by agrobacterium-mediated genetic transformation. We obtained 20 over-expression transgenic plants for each of these genes and then conducted spray inoculation testing. Results indicated that the transgenic line Os01g0138900 expressed more resistance than WT to the virulent blast strain KJ201 (Fig. 2c), suggesting that over-expression Os01g0138900 might enhancement of rice blast resistance.

### Cysteine-containing metabolities were involved in rice blast resistance

**Fig. 3:**
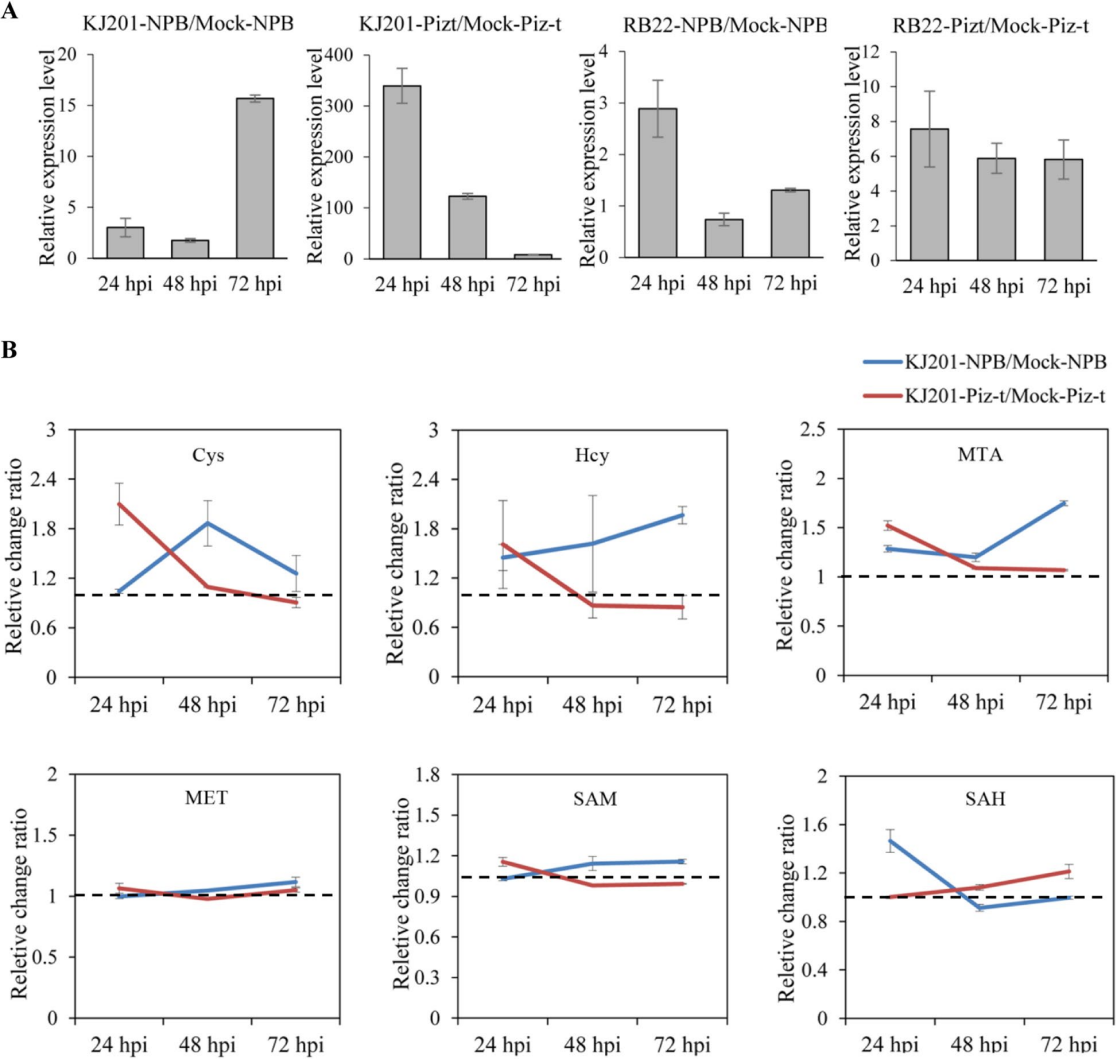
Expression pattern testing of Os04g0659300 and quantification of its downstream substances in NPB and NPB-Piz-t. **a **The expression levels of Os04g0659300 was quantified by RT-PCR. The gene expression levels in NPB and NPB-Piz-t with buffer were normalized as the calibrators. Error bars indicate standard deviations of three biological replicates. **b **The relative expression level of six metabolites. CYS, Cysteine; HCY, homocysteine; MTA, Methylthioadenosine; MET, Methionine; SAM, S-adensylmethionine; SAH, S-adenosylhomocysteine

As gi|59,800,021 is a cysteine-rich receptor-like kinases, which may play key roles in immunity, however, we didn’t obviously find that knock-out or over-expression of Os04g0659300 encoding gi|59,800,021 affected rice blast resistance. Next, we sampled the rice leaves for the gene expression level validation using quantitative real-time polymerase chain reaction (qRT-PCR), with the comparisons of KJ201-Pizt/Mock-Piz-t, RB22-Pizt/Mock-Piz-t, KJ201-NPB/Mock-NPB, and RB22-NPB/Mock-NPB at 24, 48, and 72 hpi. The qRT-PCR results indicated that compared with no obvious or inconsistent expression levels in the three susceptible groups, Os04g0659300 was greatly up-regulated in the resistance comparison of KJ201-Pizt/Mock-Piz-t at the three time-point, especially at 24 hpi (Fig. 3a), which suggests that expression of Os04g0659300 may played some roles in the resistance response.

To further confirm whether cysteine-containing substances as gi|59,800,021 was correlated with rice blast resistance, six metabolities derived from cysteine were compared in KJ201-Pizt/Mock-Piz-t and KJ201-NPB/Mock-NPB at 24, 48, and 72 hpi. Results showed that Cysteine, Homocysteine, Methylthioadenosine, Methionine, S-adensylmethionine were higher in KJ201-Pizt/Mock-Piz-t at 24 hpi, but lower at 48 and 72 hpi than in KJ201-NPB/Mock-NPB, only the S-adenosylhomocysteine had a reverse expression pattern (Fig. 3b), suggesting that some kind of on-and -off relationship exists between the former five ones and S-adenosylhomocysteine that affected the resistance response.

## Discussion

Rice blast diseases is a major factors limiting rice production, most of the major *R* genes identified to date confer race-specific resistance to their adapted pathogens, leading to their durability in the field is typically short due to mutations in the pathogen population that overcome the resistance. An alternative strategy of incorporating major R with basal resistance genes into elite cultivars to broaden or prolonged their application period is one of the most economical and environmentally friendly approaches to solve this problem.

In this study, we identified several proteins were involved into rice basal resistance, among which gi|54,290,836 is a L-Ala-D/L-amino acid epimerase, the function of this types of genes in bacteria were demonstrated to be metabolism of the murein peptide of peptidoglycan (Gulick et al. 2001; Klenchin et al. 2004). Although no peptidoglycan has been found in land plants, the ubiquity of those genes in land plants and the evidence of their expression indicate that they are functional and may play important roles in the growth and development of plants. We found that Os01g0138900 knock-out and over-expression affected rice resistance to blast. Additionally, gi|59,800,021 is acysteine-rich receptor-like kinases encoding plasma member-anchored PRRs. As PRRs recognize relatively conserved molecules from microbes, PRR-mediated resistance mechanisms have strong potential for the generation of broad-spectrum resistant cultivars in plants (Zipfel et al. 2004, 2006). Further, the thiol moieties of cysteine residues-contained in gi|59,800,021 were involved into production of reactive oxygen species (ROS), some metabolities associated with cysteine residues such as Cysteine, Homocysteine, Methylthioadenosine, Methionine, and S-adensylmethionine that immediately responded to the rice blast inoculation, suggesting that cysteine-containing may plays some role for generation of ROS by modifying the regulatory mechanisms of PTI.

Interestingly, gi|125,563,186, gi|45,736,168, and gi|401,140 that are involved in the tricarboxylic acid cycle, photosynthesis, and sucrose metabolism, respectively, whose expression patterns were complete difference, the changes of their expression levels reflected that the processes of energy distribution in the susceptible interaction. gi|125,563,186 encodes phosphoenolpyruvate carboxylase, which has a major anaplerotic role in replenishing the tricarboxylic acid cycle with intermediates to meet the demand of carbon skeletons for synthesis of organic acids and amino acids. The downregulated expression of gi|125,563,186 at 24, 48, and 72 hpi indicated that the susceptible interaction disturbed the normal life activities. Additionally, the expression levels changes of gi|45,736,168 reflected that rice blast firstly induces generation of ROS due to excess light absorption, which affected the photosynthetic apparatus, there was the possibility to down-regulate the assembly of light-harvesting complexes of PSI at 48 and 72 hpi, and to adapt plants not to absorb excess light. Accordingly, the sucrose synthase was activated at 24 hpi, but down-regulated at 48 hpi due to the damaged photosynthetic apparatus. However, the re-activated of gi|401,140 at 72 hpi might be a major physiological process that is affected under rice blast infection, and show the dynamic changes in protein expression. Otherwise, pathogens may reallocate the plant sugars for their own needs forcing the plants to modify their sugar content and triggering their defense responses during infection(Tauzin et al., 2014).

Both of gi|77,556,752 and gi|77,556,724 are pathogenesis-related protein whose important in blast-resistance has been reported (Kitajima et al., 1999). Thaumatin-like protein (gi|77,556,724) possess antifungal activities, and can be induced by biotic or abiotic factors, such as hormones, stress signal,thus they play a role in the life process and plant innate immunity. Some thaumatin-like protein family genes with antifungal potential have been used to improve plant disease resistance through genetic engineering (Yan et al. 2017).

## Conclusion

We identified eight common DEPs whose expression patterns were associated with basal resistance. Knock-out and over-expression Os01g0138900 encoding gi|54,290,836 could affected resistance to rice blast. qRT-PCR and metabolitics analysis results showed that the gi|59,800,021 and its downstream cysteine-containing substances were involved into rice blast resistance.

Further data analyses revealed several DEPs related to carbohydrate metabolism and pathogenesis that is affected under rice blast, and show the dynamic changes in expression levels. The results obtained in this study provide new targets for further gene functional analysis as well as rice blast resistance breeding.

## Materials and methods

### Plant materials

Rice (Oryza sativa L.) lines, including NPB and its transgenic line with *Piz-t* (NPB-Piz-t), and the *M. oryzae* isolates KJ201 and RB22 was used in this study (Tian et al. 2018).

### Rice blast strain and blast inoculation

Rice plants were grown in the greenhouse and kept under natural conditions about 2 ~ 3 weeks (3–4 leaves). Spores concentration in the suspension was adjusted to 5 × 105 conidia/mL. After spray-inoculated, the seedlings were maintained in the dark for 24 h at 28 °C and then kept under high humidity over 95% for about a week for symptom evaluation. Leaf tissues were collected from each rice line at 0, 24, 48, and 72 hpi and frozen in liquid nitrogen. Mock inoculated (control) plants were treated identically except that the pathogen suspension was replaced by water. The pathogen inoculation experiments were repeated three times.

### Construction of over-expression and CRISPR/Cas9 vector and genetic transformation

The over-expression and CRISPR-Cas9 of Os01g0138900, Os04g0659300, and Os04g0394200 or Os09g0315700 were obtained from the Wuhan Institute of Biotechnology, Wuhan, China. Full length cDNA fragment of these genes were PCR-amplified from cDNA prepared from NPB using KOD DNA polymerase (TOYOBO (Shanghai) Biotech Co., Ltd.) and confirmed by sequencing. CDS of Os01g0138900, Os04g0659300, and Os09g0315700 were cloned in the modified plant transformation vector pCAMBIA1300. The binary construct were confirmed by colony PCR, restriction digestion, and DNA sequencing. The confirmed clone were transformed into Agrobacterium strain EHA105 and used for genetic transformation of rice.

Guide RNA were designed to target exons of the Os01g0138900, Os04g0659300, Os04g0394200 genes using the website (https://chopchop.cbu.uib.no/). The over-expression and CRISPR vectors were transformed into NPB by Agrobacterium-mediated transformation. Individual T1 plants of over-expression and CRISPR-CAS9 were validated by sequencing the DNA products, using primers listed in Table S1.

### DNA, RNA isolation and qRT-PCR

The DNA was extracted from 10-day-old leaves, which were seeded in the 1/2 MS medium, with the 2% cetyltrimethyl ammonium bromide (CTAB) method. The RNA was extracted from rice leaves which were inoculated with the isolate KJ201 at different time points. The method referenced the nature magazine protocol (Chan et al., 2007). Primers for qRT-PCR were designed using Primer Premier 5 software. For Os04g0659300, Forward primer (5'—3'):AACGAGTGCTACGCCCGCCTCT,Reverse primer(5'—3'):GCCGCTGCTGATGTTGGTGC. The qRT-PCR was performed with Top Green qPCR SuperMix (TransGen). The rice gene Osubiquitin was used as internal control with forward primer (5'—3'):AACCAGCTGAGGCCCAAGA, and reverse primer:(5'—3')ACGATTGATTTAACCAGTCCATGA.

### Metabolite extraction and profiling analysis

We collected 50 mg samples of the rice leaf and vortex-mixed with 1.0 mL of 70% methanol for 30 s in 1.5 mL Eppendorf, overnight at 4 °C, and centrifuged for 10 min at 14,000 rpm at 4 °C. The supernatant of 120 μι was transferred into a fresh 2 mL LC–MS glass vial was analyzed using a liquid chromatography electrospray ionization tandemmass spectrometry(LC–ESI–MS/MS) system (HPLC, Shim-pack UFLC SHIMADZU CBM30A system; MS, Applied Biosystems 4500 Q TRAP) with a Waters ACQUITY UPLC BEH C8 column(2.1* 100 mm, 17 μm), operating in the positive-ion mode, and controlled by Analyst 1.5.2 software. The flow rate was 0.3 mL/min and the column was 50 C. The chromatographic mobile phase was an aqueous solution containing 0.1% formic acid (A) and acetonitrile (B) with the following gradient: 95% A gradient descent to 0% in 5 min and keep proportion for 0.4 min. The ESI source operation parameters were as follows: Curtain gas, 35 psi; Collision gas, 8 psi; Ionspray voltage:ESI + 5500 V;ESI- 4500 V; Source temperature, 350℃; Declustering Potential (DP):130; Entrance Potential (EP):10; Collision Cell Exit Potential (CXP): 10. DP and CE for individual MRM transitions were performed with further DP and CE optimization. A specific set of MRM transitions were monitored for each period according to the metabolites eluted within this period.

Standard substances were used to calculate the content through the equation of standard cure. CYS: y = 367.87x + 402.03, R^2^ = 0.9995. HCY: y = 1538x + 837.77. R^2^ = 0.9996. MTA: y = 15747x + 25,630, R^2^ = 0.9996. MET:y = 3945.7x + 18,618, R^2^ = 0.9998. SAM: y = 80.845x + 273.53, R^2^ = 0.9993. SAH:y = 336.51x + 242.74, R^2^ = 1.

### Supplementary Information

**Additional file 1.**

## Data Availability

The data that support the study are available from the corresponding author, upon reasonable request.
