# Supplementary material for: iTRAQ-based protein profiling and functional identification of four genes involved in rice basal resistance against Magnaporthe oryzae in two contrasting rice genotypes
Source: Stress Biol. 2023 Sep 12;3(1):39. doi: 10.1007/s44154-023-00118-w (PMC10497467; doi:10.1007/s44154-023-00118-w)
Supplement: Supplementary file 1 — Additional file 1. [file 44154_2023_118_MOESM1_ESM.docx]

Supplement Table 1 Specific primer pairs used for over-expression and CRISPR-CAS9 validation

| Gene Name | Forward primer (5' - 3') | Reverse primer(5' - 3') |
| --- | --- | --- |
| Os04g0659300 | CACATCGATCGCTAATCCA | GGTACACCTCCGACCTGAG |
| Os01g0138900 | CTGTAGCGGGCATCTTGTC | GAAAACGCCAAGAAAGAAA |
| Os04g0394200 | GCCAGGAACTACAGCAGTCAAC | AAGCAGCCAAGTTGAGAAGGTC |
| hyg for overexpression | ACGGTGTCGTCCATCACAGTTTGCC | ttccGGAAGTGCTTGACATTGGGGA |
